# Tubercular lymphadenitis with scrofuloderma

**DOI:** 10.11604/pamj.2024.47.78.42404

**Published:** 2024-02-20

**Authors:** Ashwin Karnan

**Affiliations:** 1 Department of Respiratory Medicine, Jawaharlal Nehru Medical College, Datta Meghe Institute of Higher Education and Research, Sawangi (Meghe), Wardha, Maharashtra, India

**Keywords:** Tuberculosis, granuloma, lymph node, Mantoux test

## Image in medicine

A 27-year-old male presented to the outpatient department with complaints of multiple swelling in the neck for the past 6 months associated with evening rise of temperature, loss of weight, and loss of appetite with no significant past and personal history. On examination of the neck, there were multiple, hard, mobile, discrete, non-tender, cervical lymph nodes of size 2-2.5cm with no active discharge and overlying skin showing signs of healed ulceration. The tuberculin skin test was positive, showing an induration of 20mm. Fine needle aspiration cytology from the lymph node showed caseating granulomatous inflammation suggestive of tuberculosis. A dermatologist's opinion was taken and a biopsy from the overlying skin showed central necrosis with peripheral granuloma formation suggestive of scrofuloderma. The patient was started on anti-tubercular treatment and at one month follow up there was regression of lymph nodes. Scrofuloderma is one of the cutaneous manifestations of tuberculosis, accounting for approximately 2% of extrapulmonary tuberculosis. It is also called tubercular colliquative cutis and occurs from the direct extent of disease from deep structure for example: lymph node, joint, bone, or epididymis. It is mostly seen in the adolescent age group and commonly involves the neck, axilla, and groin. They may be single or multiple. Treatment is therapy with isoniazid, rifampin, ethambutol and pyrazinamide for 9-12 months.

**Figure 1:**
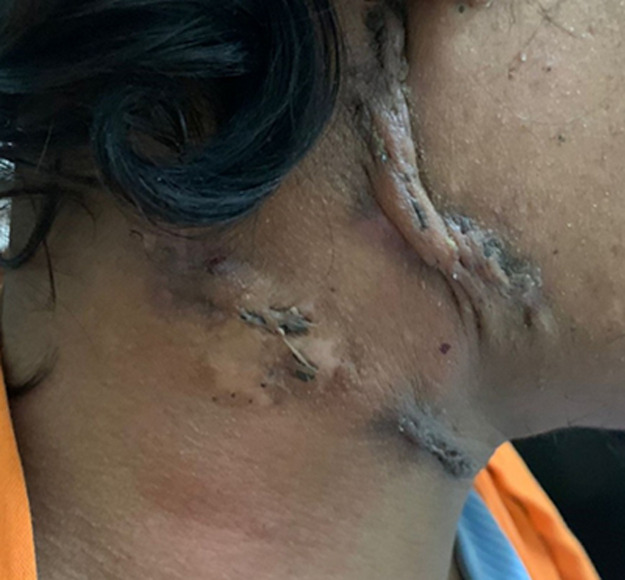
multiple enlarged right cervical lymph nodes with overlying skin showing healed ulcers and hypertrophied scars

